# Supplementary material for: Metabolomic Profiling in Patients with Heart Failure and Exercise Intolerance: Kynurenine as a Potential Biomarker
Source: Cells. 2022 May 18;11(10):1674. doi: 10.3390/cells11101674 (PMC9139290; doi:10.3390/cells11101674)
Supplement: Supplementary file 1 [file cells-11-01674-s001.zip › cells-1682441-supplementary.pdf]

Metabolomic profiling in heart failure, exercise intolerance, and reduced muscle endurance: Kynurenine as a potential biomarker

Tarek Bekfani MD[1,2], Mohamed Bekhite PhD[2], Sophie Neugebauer PhD[3], Steffen Derlien PhD[4], Ali Hamadanchi MD[2], Jenny Nisser M.A.[4], Marion S. Hilse MD[2], Daniela Haase PhD[2], Tom Kretzschmar[2], Mei-Fang Wu PhD[2], Michael Lichtenauer MD[5], Michael Kiehntopf MD[3], Stephan von Haehling MD, PhD[6], Peter Schlattmann MD[7], Gabriele Lehmann MD[8], Marcus Franz MD[2], Sven Möbius-Winkler MD[2], P. Christian Schulze MD, PhD[2]

1 Department of Internal Medicine I, Division of Cardiology, Angiology and Intensive Medical Care, University Hospital Magdeburg, Otto von Guericke-University, Magdeburg, Germany

2 Department of Internal Medicine I, Division of Cardiology, Angiology and Intensive Medical Care, University Hospital Jena, Friedrich-Schiller-University, Jena, Germany

3 Department of Clinical Chemistry and Laboratory Diagnostics, Jena University Hospital, Jena, Germany

4 Institute of Physiotherapy, University Hospital Jena, Friedrich-Schiller-University, Jena, Germany

5 Clinic of Internal Medicine II, Department of Cardiology, Paracelsus Medical University of Salzburg, Austria

6 University of Göttingen Medical Center, Department of Cardiology and Pneumology, Göttingen, Germany and German Center for Cardiovascular Research (DZHK), partner site Göttingen, Germany

7 Institute for Medical Statistics, Computer Science and Data Science (IMSID), Jena University Hospital, Jena, Germany

8 Department of Internal Medicine III, Division of Endocrinology, Nephrology and Rheumatology, University Hospital Jena, Friedrich-Schiller-University, Jena, Germany

Address for correspondence:
P. Christian Schulze, MD; PhD
Department of Internal Medicine I
Division of Cardiology
University Hospital Jena
Am Klinikum 1, 07747 Jena
Germany
Tel.: +49 3641 932 4101
Fax: +49 3641 932 4102
E-Mail: christian.schulze@med.uni-jena.de

SUPPLEMENTARY DATA

Table S1: Concentration of amino acids and biogenic amines in serum in patients with HF and in HC.

	**HFpEF**<br><br>**N=17**	**HFrEF**<br><br>**N=18**	**HC**<br><br>**N=20**	**p-value**
**Alanine** (µM)	430±174	462±162	549±98	0.043
**Asparagine** (µM)	47.40 [42.10-59.30]	49.70 [41.30-55.77]	52.0 [48.0-78.55]	0.08
**Glutamate** (µM)	55.0 [51.05-81. 30]*	66.50 [50.0-83.63] †	158 [144-204]	<0.001
**Glycine** (µM)	208 [149-288]*	206 [110-249] †	405 [281-537]	0.001
**Ornithine** (µM)	113 [93.40-123]*	111 [87.8-141] †	166 [134-190]	0.001
**Phenylalanine** (µM)	73±9	81±12 †	65±12	<0.001
**Tryptophan** (µM)	66±15	72±12	79±17	0.047
**Valine** (µM)	295±93*	308±72 †	193±59	<0.001
**Essential amino acids** (µM)	140±37.2	143±29.5	125±29.5	0.19
**Kynurenine** (µM)	3.7±0.9*	4.7±1.7 †	1.6±0.9	< 0.001

∗: p<0.0167 in comparison between HFpEF and HC.

†: p<0.0167 in comparison between HFrEF and HC.

HC: healthy controls, HFpEF: Heart failure with preserved ejection fraction, HFrEF: Heart failure with reduced ejection fraction,

Table S2: Concentration of ACs in serum in patients with HF and in HC.

	HFpEF	HFrEF	HC	p-Value
**C0** (µM)	36.50 [31.0-40.40]*	40.60 [35.58-44.78] †	28.40 [25.40-34.35]	<0.001
**Short-chain ACs** (µM)	0.19 [0.07-0.30]	0.24 [0.10-0.39]	0.24 [0.20-0.31]	0.26
**Medium-chain ACs** (µM)	0.38 [0.14-1.47]*	0.32 [0.14-1.64] †	0.12 [0.10-0.13]	<0.001
**Long-chain ACs** (µM)	0.06 [0.05-0.06]*	0.06 [0.05-0.08] †	0.04 [0.03-0.05]	<0.001
**Unsaturated ACs** (µM)	0.08 [0.06-0.10]*	0.10 [0.05-0.12] †	0.05 [0.05-0.06]	<0.001
**C10** (µM)	0.96 [0.27-7.21]*	0.67 [0.29-8.07] †	0.23 [0.18-0.28]	0.001
**C10:1** (µM)	0.26 [0.15-0.36]*	0.28 [0.12-0.47] †	0.13 [0.09-0.16]	0.003
**C12:1** (µM)	0.15 [0.12-0.20]*	0.16 [0.13-0.27] †	0.11 [0.09-0.13]	<0.001
**C14:1** (µM)	0.12 [0.07-0.16]*	0.13 [0.07-0.19] †	0.07 [0.06-0.09]	0.001
**C14:1-(OH)** (µM)	0.02 [0.01-0.03]*	0.03 [0.02-0.04] †	0.01 [0.01-0.02]	<0.001
**C14:2-(OH)** (µM)	0.02 [0.01-0.02]	0.02 [0.01-0.03] †	0.01 [0.01-0.01]	0.016
**C16:1** (µM)	0.04 [0.03-0.05]	0.05 [0.04-0.07] †	0.03 [0.03-0.04]	0.005
**C16:1-(OH)** (µM)	0.02 [0.01-0.02]*	0.02 [0.01-0.02]	0.01 [0.01-0.01]	0.007
**C16:2** (µM)	0.02 [0.02-0.03]*	0.02 [0.01-0.04]	0.01 [0.01-0.02]	0.025
**C16:2-(OH)** (µM)	0.02 [0.01-0.13]*	0.02 [0.01-0.16] †	0.01 [0.01-0.02]	0.001
**C18:1** (µM)	0.19 [0.14-0.20]*	0.20 [0.13-0.27] †	0.12 [0.09-0.16]	0.004
**C18:1-(OH)** (µM)	0.03 [0.02-0.13]*	0.04 [0.03-0.16] †	0.02 [0.02-0.03]	<0.001
**C5-M-DC** (µM)	0.07 [0.02-0.12]*	0.05 [0.02-0.12] †	0.02 [0.02-0.03]	<0.001
**C6:1** (µM)	0.03 [0.01-0.07]	0.03 [0.02-0.06] †	0.02 [0.01-0.02]	0.021

∗: p<0.0167 in comparison between HFpEF and HC. †: p<0.0167 in comparison between HFrEF and HC.

AC = acylcarnitine. Cx:y: where x is the number of carbons in the fatty acid side chain; y is the number of double bonds in the fatty acid side chain; DC: decarboxyl; M: methyl; ns: not significant; OH: hydroxyl, HC: healthy controls, HFpEF: Heart failure with preserved ejection fraction, HFrEF: Heart failure with reduced ejection fraction,

Table S3: Concentration of PCs and Lyso-PCs in serum in patients with HF and in HC.

	**HFpEF**	**HFrEF**	**HC**	**p-value**
**PC aa C32:0 (µM)**	11.9±2.9*	13.9±3.7	15.3±3.2	0.009
**PC aa C32:3 (µM)**	0.4±0.1*	0.4±0.2†	0.7±0.2	<0.0001
**PC aa C34:2 (µM)**	376±107*	425±182†	666±101	<0.0001
**PC aa C34:3 (µM)**	15.4±4.9*	16.7±8.1†	25.7±9.9	<0.0001
**PC aa C36:0 (µM)**	2.4±0.8*	2.6±1.3†	3.9±1.0	<0.0001
**PC aa C36:1 (µM)**	38.0±19.1*	34.2±8.9	24.0±7.9	0.004
**PC aa C36:2 (µM)**	218.8±42.1*	223.8±74.0†	308.8±55.8	<0.0001
**PC aa C36:3 (µM)**	122.2±28.9*	132.3±44.8†	175.7±28.7	<0.0001
**PC aa C36:4 (µM)**	171.8±39.6*	188.4±58.8†	239.8±36.8	<0.0001
**PC aa C36:6 (µM)**	0.9±0.3	0.9±0.3	1.3±0.6	0.04
**PC aa C38:0 (µM)**	2.2±0.5	2.3±0.8	2.8±1.0	0.04
**PC aa C40:2 (µM)**	0.20 [0.18-0.27]*	0.23 [0.18-0.35]	0.32 [0.22-0.36]	0.007
**PC aa C40:3 (µM)**	0.50 [0.39-0.56]*	0.51 [0.37-0.66] †	0.64 [0.60-0.90]	0.001
**PC aa C42:4 (µM)**	0.08±0.05*	0.07±0.04	0.04±0.03	0.002
**PC aa C42:5 (µM)**	0.3±0.1*	0.4±0.2	0.5±0.2	0.01
**LysoPC a 16:0 (µM)**	82.5±16.8*	87.8±31.8	109.4±25.2	0.005
**LysoPC a 17:0 (µM)**	1.7±0.4	1.6±0.6	2.0±0.5	0.04
**LysoPC a 18:0 (µM)**	23.4±3.9	22.2±7.0	27.4±5.7	0.02

**LysoPC a 26:0 (µM)**	0.09±0.05	0.08±0.04	0.06±0.03	0.02
**PC ae C32:1 (µM)**	2.3±0.5*	2.4±0.7†	3.1±0.9	0.001
**PC ae C34:1 (µM)**	8.0±2.0	8.5±2.9	10.2±2.3	<0.02
**PC ae C34:2 (µM)**	9.0±1.7*	8.7±2.9†	11.9±2.5	<0.0001
**PC ae C34:3 (µM)**	5.3±1.2*	4.9±1.3†	7.7±2.2	<0.0001
**PC ae C36:0 (µM)**	0.8±0.3*	0.9±0.4	1.2±0.3	0.002
**PC ae C36:2 (µM)**	11.2±2.6*	11.1±3.2†	14.4±2.7	0.001
**PC ae C36:3 (µM)**	5.5±1.2	5.4±1.6†	6.8±1.3	0.004
**PC ae C36:5 (µM)**	8.9±2.2*	9.6±2.9	11.6±2.9	0.008
**PC ae C38:0 (µM)**	1.7±0.5	1.7±0.5	2.3±0.8	0.008
**PC ae C38:3 (µM)**	3.2±0.8	3.3±1.1	4.0±0.8	0.02
**PC ae C40:1 (µM)**	0.9±0.3*	0.9±0.2†	1.3±0.4	0.002
**PC ae C40:3 (µM)**	0.6±0.2	0.6±0.2†	0.8±0.2	0.006
**PC ae C40:4 (µM)**	1.5±0.4*	1.6±0.5†	2.1±0.5	0.002
**PC ae C40:5 (µM)**	2.6±0.6*	2.9±1.0	3.6±0.7	0.001
**PC ae C40:6 (µM)**	3.7±0.7	4.0±1.4	4.8±1.4	0.02
**PC ae C42:0 (µM)**	0.6±0.1	0.6±0.2	0.7±0.1	<0.05
**PC ae C42:1 (µM)**	0.2±0.1*	0.2±0.1	0.3±0.1	0.005
**PC ae C42:3 (µM)**	0.5±0.1	0.5±0.1†	0.6±0.1	0.003

**PC ae C42:4 (μM)**	0.6±0.2	0.5±0.2[†]	0.7±0.2	0.002
**PC ae C42:5 (μM)**	1.6±0.2[*]	1.7±0.3[†]	1.9±0.3	<0.0001

*: p<0.0167 in comparison between HFpEF and HC. †: p<0.0167 in comparison between HFrEF and HC.

a = acyl, aa = diacyl, ae =acyl-alkyl, Cx:y = where x is the number of carbons in the fatty acid side chain; y is the number of double bonds in the fatty acid side chain, OH =hydroxyl, LysoPC: Lysophosphatidylcholines,

PC = phosphatidylcholine.

Table S4: Concentrations of SMs in patients with HF and in HC.

	HFpEF	HFrEF	HC	p-value
**SM (OH) C14:1 (μM)**	8.5±3.0	9.1±4.8	11.7±3.4	0.03
**SM (OH) C22:1 (μM)**	10.5±3.2	10.3±2.2	12.4±2.1	0.02
**SM C16:0 (μM)**	122±29*	129±40†	166±27	<0.0001
**SM C16:1 (μM)**	18.6±5.0*	18.9±5.9†	24.6±3.9	0.001
**SM C20:2 (μM)**	0.41±0.12*	0.38±0.21†	0.66±0.17	<0.0001
**SM C24:0 (μM)**	17.0±4.5*	17.2±4.6†	22.2±3.4	<0.0001
**SM C24:1 (μM)**	54.0±11.3*	56.3±12.5†	67.3±10.0	0.001
**SM C26:0 (μM)**	0.11 [0.08-0.15]*	0.11 [0.08-0.15]	0.14 [0.11-0.22]	0.04

*: p<0.0167 in comparison between HFpEF and HC.

†: p<0.0167 in comparison between HFrEF and HC.

Cx:y = where x is the number of carbons in the fatty acid side chain; y is the number of double bonds in the fatty acid side chain, OH =hydroxyl, SM: sphingomyelin, HC: healthy controls, HFpEF: Heart failure with preserved ejection fraction, HFrEF: Heart failure with reduced ejection fraction.
